# Optical Coherence Tomography Angiography in Retinal Vascular Diseases and Choroidal Neovascularization

**DOI:** 10.1155/2015/343515

**Published:** 2015-09-27

**Authors:** Rodolfo Mastropasqua, Luca Di Antonio, Silvio Di Staso, Luca Agnifili, Angela Di Gregorio, Marco Ciancaglini, Leonardo Mastropasqua

**Affiliations:** ^1^Ophthalmology Unit, Department of Neurological, Neuropsychological, Morphological and Movement Sciences, University of Verona, 37100 Verona, Italy; ^2^Ophthalmology Clinic, Department of Medicine and Aging Science, University G. d'Annunzio of Chieti-Pescara, 66100 Chieti, Italy; ^3^Eye Clinic, Department of Life, Health and Environmental Sciences, University of L'Aquila, 67100 L'Aquila, Italy

## Abstract

*Purpose*. To assess the ability of optical coherence tomography-angiography (OCT-A) to show and analyze retinal vascular patterns and the choroidal neovascularization (CNV) in retinal vascular diseases.* Methods*. Seven eyes of seven consecutive patients with retinal vascular diseases were examined. Two healthy subjects served as controls. All eyes were scanned with the SD-OCT XR Avanti (Optovue Inc, Fremont CA, USA). Split spectrum amplitude decorrelation angiography algorithm was used to identify the blood flow within the tissue. Fluorescein angiography (FA) and indocyanine green angiography (ICGA) with Spectralis HRA + OCT (Heidelberg Engineering GmbH) were performed.* Results*. In healthy subjects OCT-A visualized major macular vessels and detailed capillary networks around the foveal avascular zone. Patients were affected with myopic CNV (2 eyes), age-related macular degeneration related (2), branch retinal vein occlusion (BRVO) (2), and branch retinal artery occlusion (BRAO) (1). OCT-A images provided distinct vascular patterns, distinguishing perfused and nonperfused areas in BRVO and BRAO and recognizing the presence, location, and size of CNV.* Conclusions*. OCT-A provides detailed images of retinal vascular plexuses and quantitative data of pathologic structures. Further studies are warranted to define the role of OCT-A in the assessment of retinovascular diseases, with respect to conventional FA and ICG-A.

## 1. Introduction

Fifty years have passed since Novotny and Alvis performed the first fluorescein angiography (FA). This invasive method uses intravenous fluorescein to produce fluorescence images of circulating blood in the human retina [1]. For many years this procedure has been considered the gold standard for imaging the retinal vasculature network [2].

From its commercialization, both time and spectral domain (SD) optical coherence tomography (OCT) modalities have dramatically changed the daily clinical practice in ophthalmology [3, 4]. With the introduction of SD-OCT, acquisition time decreased while the imaging resolution greatly improved. Thus it was possible to generate clinically useful cross-sectional and 3-dimensional (3D) images of the retinal layers [5]. A limit remains in that this imaging modality cannot visualize and thus cannot provide functional information of retinal microcirculation.

Recently, a novel dyeless method of microvasculature imaging called OCT angiography (OCT-A) was introduced. Several prototypes used the normal movement of the red blood cells in the retinal capillaries as intrinsic contrast medium to generate flow imaging. The phase-based and amplitude-based modalities showed feasibility of rendering deeper structural details of retinal and choroidal microvascular structures when motion-based contrast techniques are used [6–9]. Three examples that are currently in use are as follows: Phase Variance OCT (PV-OCT), Phase Contrast OCT (PC-OCT), and Split Spectrum Amplitude Decorrelation Angiography (SSADA) (Angio-OCT). These methods were implemented using both SD and swept-source OCT (SS-OCT) imaging systems in order to detect the transverse and axial flow thus differing from Doppler OCT that measures just the axial velocity. Pilot studies that investigated these OCT systems in patients with age-related macular degeneration and glaucoma [10–12] confirmed their utility.

Amplitude-based OCT signal analysis may be advantageous for ophthalmic imaging since it uses a speckle variance method that does not suffer from phase noise artifacts and does not require complex phase correction methods [8]. Both phase-based and amplitude-based OCT provide noninvasive visualization of both larger blood vessels and capillary networks in the retina and choroid [6, 8]. The results obtained with these modalities were comparable to currently used invasive angiographic imaging [6, 8] in addition to being able to separately characterize features of the superficial and deep vascular plexuses, which cannot be distinguished in FA [9]. The aim of this study was to evaluate the ability of OCT-A for imaging retina of subjects affected with vascular diseases to assess perfused and nonperfused areas and choroidal neovascularization (CNV), using XR Avanti AngioVue OCT (Optovue, Inc, Fremont CA, USA).

## 2. Materials and Methods

This prospective unmasked study adhered to the tenets of the Declaration of Helsinki, and informed consent was obtained from all patients prior to their enrolment. Our local Ethics Board was notified and stated that their approval was not necessary. Seven eyes of seven consecutive Caucasian patients (four males and three females) ranging in age from 54 to 64 years (mean 59.1 years), referred to the Ophthalmic Clinic of University Chieti-Pescara, Italy, for the presence of retinal vascular diseases from October 1 to 31, 2014, were examined. Two healthy subjects with no significant medical history and no signs or symptoms of retinal vascular disease were included as controls. FA was performed in all subjects with the Spectralis HRA + OCT (Heidelberg Engineering GmbH). Indocyanine green angiography (ICGA) was performed only in patients with suspected choroidal neovascularization (CNV). All eyes were also scanned with the SD-OCT XR Avanti (Optovue Inc, Fremont CA, USA), using the following settings: high speed (70,000 A-scans/seconds), 840 nm wavelength (band-width 45 nm), and an axial resolution of 5 *μ*m. Each B-scan contained 216 A-scans. The scanning procedure was described in detail elsewhere [13]. The SSADA algorithm was used to distinguish between static and nonstatic tissue. This algorithm identifies blood flow by calculating the decorrelation of signal amplitude from consecutive B-scans performed at the same retinal acquisition plane.

Decorrelation of OCT signal amplitude between B-scans taken at the same nominal position could be caused by flow, bulk tissue motion, scanner position error, and background noise. To enhance true flow in the images and improve the signal-to-noise ratio for flow detection, the decorrelation due to bulk motion and background noise were eliminated. The SSADA data was separated into retinal and choroidal regions with the dividing boundary set at the retinal pigment epithelium (RPE). The depth (*Z* position) of the highly reflective RPE was identified through the analysis of the reflectance and reflectance gradient profiles in depth [14]. The region above the RPE was the retinal layer and the region below was the choroidal layer. Since the retina is a laminar structure with a stratified blood supply,* en face X-Y* projection angiograms were produced by selecting the maximum decorrelation value along the axial (*Z*) direction in each layer. Segmentation of the retina in specific layers provided a simple en face visualization of the corresponding vascular supply for that layer.

Automatic segmentation was used to identify retinal layers. When a retinal pathology was present (i.e., presence of subretinal fluid or serous pigment epithelial detachment), manual correct of the segmentation was necessary in order to avoid both image processing software and segmentation errors [9]. The allowable field of view (FOV) in the retina SSADA scans is 2 × 2, 3 × 3, 6 × 6, and 8 × 8 mm. A 3 × 3 mm FOV is the recommended default size for visualization of retinal capillaries. The 6 × 6 or 8 × 8 mm scans are the recommended scans for performing large area scans of the retina.

## 3. Results

**Figure 1:**
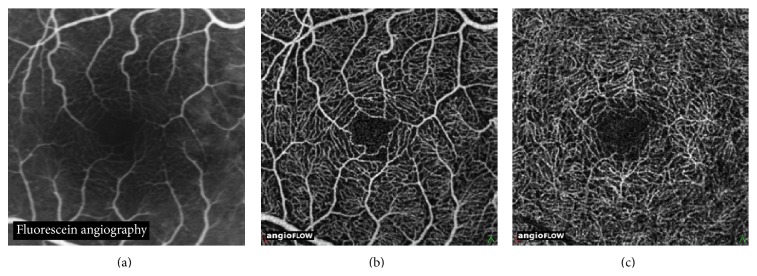
Fluorescein angiography image of the central macula in a healthy subject (a). OCT angiography image (3 × 3 mm) of the superficial vascular plexus (b) showing the vascular centripetal distribution “web-like shape” towards the fovea. OCT angiography image (3 × 3 mm) of the deep vascular plexus (c) showing a close-knit pattern of vessels around the foveal avascular zone. OCT angiography identifies more about details of the capillary beds (superficial and deep) than standard Fluorescein Angiography.

In healthy subjects OCT-A visualized major macular vessels and detailed capillary networks around the foveal avascular zone. The vascular network was similar to those reported using FA (Figure 1(a)). Notably, OCT-A revealed more capillary details compared to FA in the superficial and, especially, in the deep retinal layer (Figures 1(b) and 1(c)).

**Figure 2:**
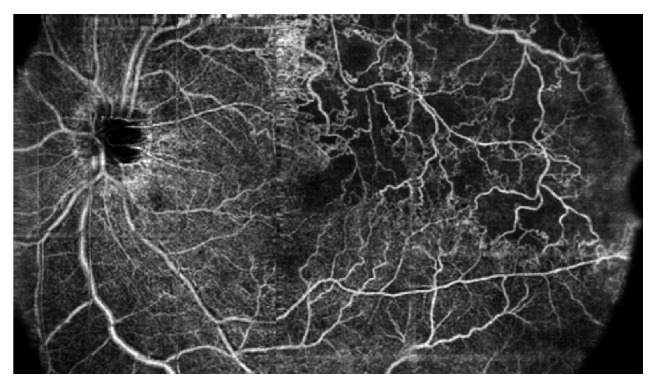
OCT angiography (composition map of three partially overlapping 8 × 8 mm scans) of a 57-year-old woman with branch retinal vein occlusion (BRVO) showing enlargement of foveal avascular zone and retinal nonperfused area (capillary drop-out) at the posterior pole and at the mid periphery with the development of collaterals.

**Figure 3:**
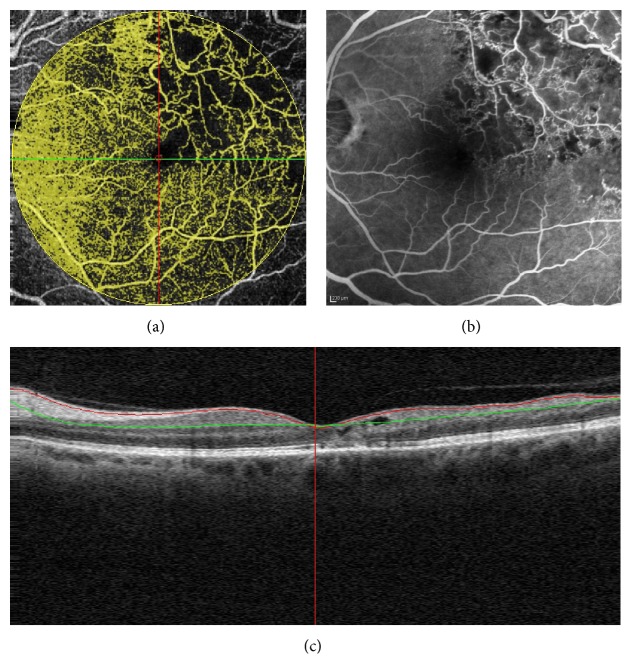
Branch retinal vein occlusion (BRVO) of a 53-year-old woman: OCT angiography (8 × 8 mm) showing perfused and nonperfused flow areas of the retina (a) due to capillary drop-out seen in fluorescein angiography (b). Longitudinal B-scan demonstrating the reference lines of the superficial plane (c).

Patients 1 and 2 were affected with ischemic branch retinal vein occlusion (BRVO). A composite map composed of three 8 × 8 mm scans from patient 1 and an 8 × 8 mm OCT-A scan from patient 2 are shown in Figures 2 and 3(b), respectively. In both cases, OCT-A distinguished perfused and nonperfused areas located in both the posterior pole and mid periphery (Figures 2 and 3).

**Figure 4:**
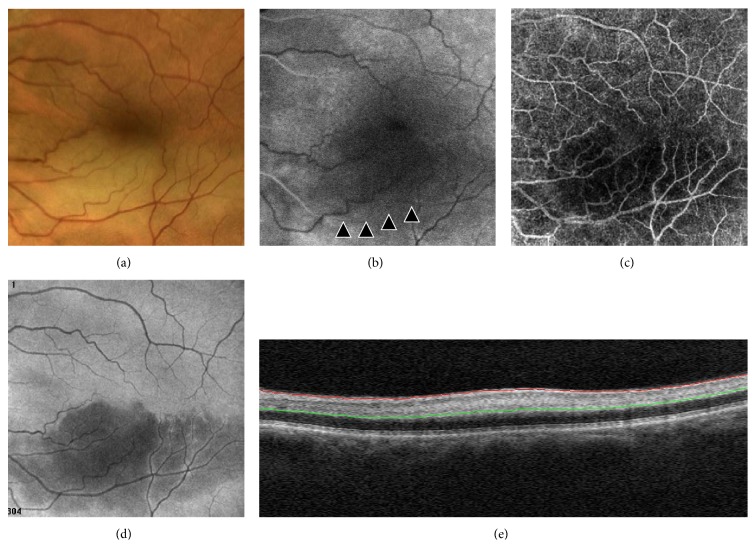
Branch retinal artery occlusion (BRAO) of a 62-year-old man showing a band of retinal whitening extending inferior-temporally from the disc in a color image (a). Delayed filling (about 17 sec) of the arteriole (arrowheads) during the arterial phase in fluorescein angiography (b). Low flow area in OCT-angiography (6 × 6 mm) image (c). Area of hyporeflectivity in high-resolution en face projection adapted to the retinal pigment epithelium surface (d). Focal thickening and hyperreflectivity due to intraretinal edema in the reference plane of B-scan (e).

Patient 3 was affected with branch retinal artery occlusion (BRAO). The main finding was the low flow area in OCT-A, which correlated with the area of hypofluorescence in FA due to retinal artery hypoperfusion. Moreover, the en face image adapted to the RPE surface showed the presence of ischemia-induced area of hyporeflectivity and longitudinal B-scan revealed focal thickening and hyperreflectivity within both inner and deep capillary plexuses (Figure 4).

**Figure 5:**
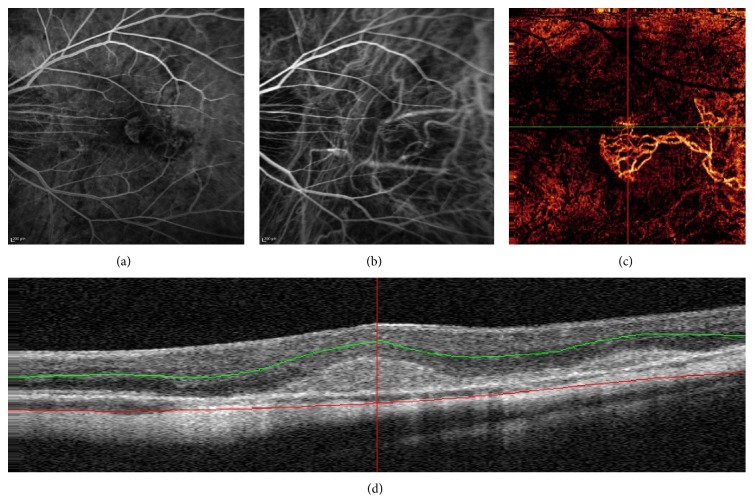
Simultaneous fluorescein angiography (a) and Indocyanine green angiography arteriovenous phase (b) showing myopic choroidal neovascularization. Color-mode OCT angiography (3 × 3 mm) image (c) shows both feeder vessels and neovascular network of myopic choroidal neovascularization. B-scan (d) showing reference planes at the level of the outer retina.

**Figure 6:**
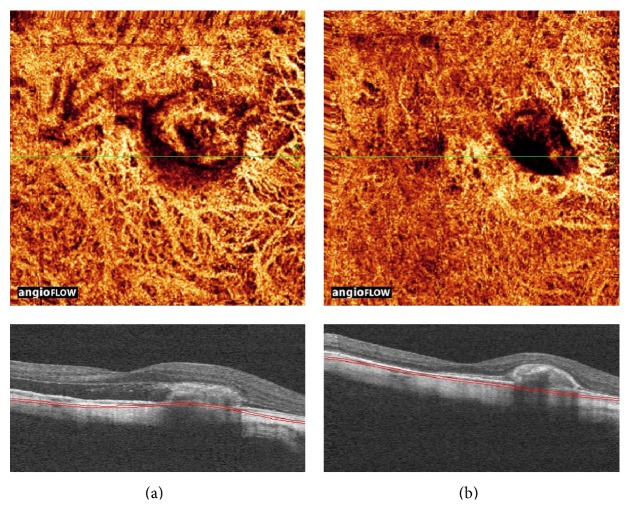
Angioretina color-mode change analysis. (a) Baseline showing neovascular network of myopic choroidal neovascularization (top) and the presence of subretinal fluid in longitudinal B-scan (bottom). (b) Follow-up showing reduction of both flow and size of neovascular complex after intravitreal injection of anti-VEGF (top) and resolution of SRF in longitudinal B-scan (bottom).

In patient 4, the color-mode OCT-A aided in detecting the vascular network of myopic CNV and in defining specific morphological features (Figure 5). The entire vascular pattern was seen with enhanced details compared to simultaneous FA and ICGA frames. Color-mode OCT-A in patient 5, affected by myopic CNV, showed the response to therapy with intravitreal antivascular endothelial growth factor medications (Figure 6). Analysis of OCT-A changes indicated a reduction in size and flow of CNV and the resolution of subretinal fluid observed in B-scan.

**Figure 7:**
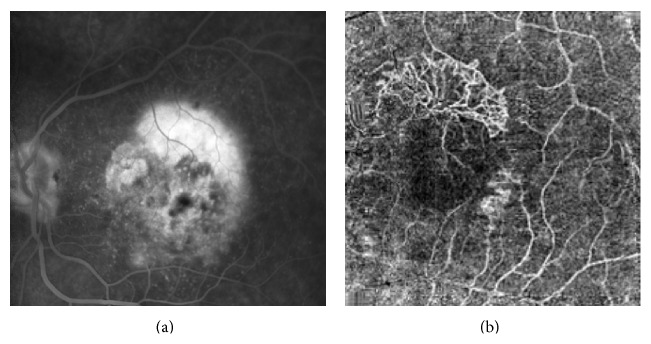
Fluorescein angiography (a) shows late leakage from classical choroidal neovascularization. OCT angiography (6 × 6 mm) and (b) enhances choroidal new vessels as high-flow network “umbrella like-shape” above retinal pigment epithelium (RPE).

**Figure 8:**
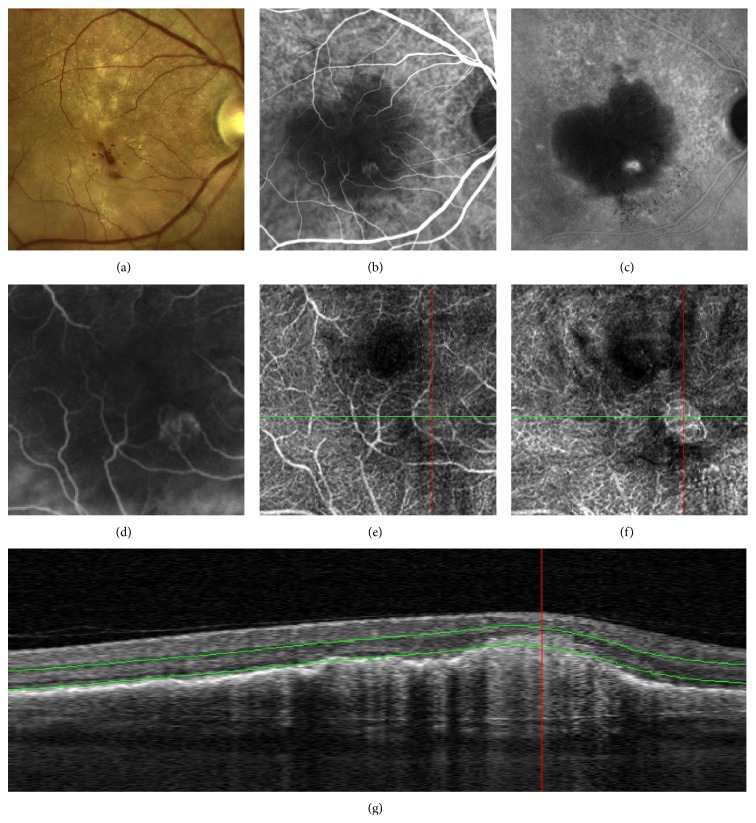
Multimodal retinal imaging of retinal angiomatous proliferation (RAP): color picture (a) showing intraretinal hemorrhages and exudates. Early phase of indocyanine green angiography (ICG-A) (b) revealing typical features such as feeding artery, a focal hyperfluorescence, and draining vein, seen better in the enlarged view (d). Late phase of ICG-A (c) showing a hot-spot within a serous pigment epithelial detachment (PED). OCT-Angiography scan (3 × 3 mm) (e) adapted to inner vascular plexus showing a subtle glomerular network. OCT angiography scan (3 × 3 mm) (f) highlighting glomerular high flow arising from deep capillary plexus. B-scan (g) demonstrating the reference plane at the level of deep capillary plexus.

Patients 6 and 7 were affected with CNV secondary to age-related macular degeneration (AMD). Classic CNV (Figure 7) focused on the outer capillary plexus and above the RPE. Retinal angiomatous proliferation (Figure 8) was detected at the level of the deep capillary plexus. By using OCT-A, CNV was clearly recognizable and the rim and area of neovascularization were more easily detectable compared to FA and ICGA.

## 4. Discussion

This study aimed at examining the ability of OCT-A, a promising and noninvasive imaging modality, to image the vascular modifications within the inner and outer retinal layers in several retinovascular diseases. Currently, FA and ICGA are considered the gold standard for defining functional and morphological features in patients affected with ocular vascular diseases. However, both techniques are invasive since they require intravenous dye injection and are bothersome for some patients who complain of side effects that range from nausea/vomiting to serious anaphylactic reactions (although the latter is rare). Moreover, FA can only show vessels in a nearly transparent structure with a thickness that is on the order of only hundreds of micrometers. This allows a good visualization of the superficial capillary network of the retina but not of the deep capillary layer [15, 16]. On the other hand, OCT angiography obtained with the SSADA algorithm provides images of both the superficial and deep retinal vascular plexuses.

In the present study both OCT-A and FA provided clinically useful images of the inner retinal vascular plexus. This was in accordance with results of Imai et al. [17] who used swept-source OCT in patients with BRVO, reporting a good agreement between FA and OCT-A in assessing the area and colocalization of nonperfused area. The authors concluded that OCT angiography was a valuable alternative imaging modality for studying ischemic diseases in both the diagnostic phase and during follow-up. Currently, the role of the outer retinal plexus in retinal diseases is unclear. In fact, vascular diseases could affect inner and outer plexuses differently. In support of this, vein occlusions and inflammatory vascular diseases may be selectively involved, as suggested by modifications of middle layers in OCT B-scans.

One of the most promising fields for the application of OCT-A is CNV imaging. Other structural OCT imaging modalities cannot directly identify the CNV structure, but only the presence of changes such as abnormal tissue above or below the RPE. Therefore, FA and ICGA are still needed in the initial diagnosis of neovascular AMD.

The present study showed that OCT-A was also valuable in imaging and quantifying CNV in myopia and AMD since the neovascularization was successfully identified in all patients presenting this lesion. The SSADA algorithm was crucial in differentiating CNV from the surrounding outer retinal tissue, RPE, and hemorrhages. This confirmed the preliminary data reported by Jja et al. in a swept-source OCT prototype study [10]. Notably, CNV vascular network patterns were more distinct in OCT-A scans compared to FA and ICGA, particularly in terms of location, size, and presence of feeder vessels.

It is important to keep in mind that subretinal and surrounding CNV fluid appeared as a low flow area in OCT-A (because SSADA identifies only moving flow), whereas it produced a hyperfluorescent pattern in FA; where the fluid outside of the blood vessels generates dye leakage Overall, OCT-A was not completely comparable to FA and ICGA angiograms. In fact, OCT-A provided static images of the vascular flow, without providing dynamic information, which was conversely provided by classical ocular angiographies with dyes. However, the integration of structural and quantitative data obtained with OCT has the potential to be a new useful tool for studying retinal vascular diseases.

Although this pilot study has the weaknesses of limited sample size it showed the potential of OCT-A for providing quantitative data for diagnosis and follow-up of different retinochoroidal diseases without the use of intravenous dyes. The first limit of this new technique was that the field of view (3 × 3 mm, 6 × 6 mm and 8 × 8 mm) was smaller than conventional FA. In the future the FOV should increase as scan speeds increase. The second limit was that a precise fixation was required in order to obtain good images, so this technique was not reproducible in patients with low vision. Further investigations will be needed to validate the clinical application of this novel method.
